# TrkB Signaling Influences Gene Expression in Cortistatin-Expressing Interneurons

**DOI:** 10.1523/ENEURO.0310-19.2019

**Published:** 2020-02-06

**Authors:** Kristen R. Maynard, Alisha Kardian, Julia L. Hill, Yishan Mai, Brianna Barry, Henry L. Hallock, Andrew E. Jaffe, Keri Martinowich

**Affiliations:** 1 Lieber Institute for Brain Development, Johns Hopkins Medical Campus, Baltimore, Maryland 21205; 2 Department of Mental Health, Johns Hopkins University, Baltimore, Maryland 21205; 3 Department of Biostatistics, Johns Hopkins Bloomberg School of Public Health, Baltimore, Maryland 21205; 4 Department of Psychiatry and Behavioral Sciences, Johns Hopkins University School of Medicine, Baltimore, Maryland 21287; 5 The Solomon H. Snyder Department of Neuroscience, Johns Hopkins University School of Medicine, Baltimore, Maryland 21205

**Keywords:** ASD, BDNF-TrkB, cortistatin, epilepsy, inhibitory interneurons, Ribotag

## Abstract

Brain-derived neurotrophic factor (BDNF) signals through its cognate receptor tropomyosin receptor kinase B (TrkB) to promote the function of several classes of inhibitory interneurons. We previously reported that loss of BDNF–TrkB signaling in cortistatin (Cort)-expressing interneurons leads to behavioral hyperactivity and spontaneous seizures in mice. We performed bulk RNA sequencing (RNA-seq) from the cortex of mice with disruption of BDNF–TrkB signaling in cortistatin interneurons, and identified differential expression of genes important for excitatory neuron function. Using translating ribosome affinity purification and RNA-seq, we define a molecular profile for Cort-expressing inhibitory neurons and subsequently compare the translatome of normal and TrkB-depleted Cort neurons, revealing alterations in calcium signaling and axon development. Several of the genes enriched in Cort neurons and differentially expressed in TrkB-depleted neurons are also implicated in autism and epilepsy. Our findings highlight TrkB-dependent molecular pathways as critical for the maturation of inhibitory interneurons and support the hypothesis that loss of BDNF signaling in Cort interneurons leads to altered excitatory/inhibitory balance.

## Significance Statement

Mounting evidence suggests that brain-derived neurotrophic factor (BDNF) signals through its receptor TrkB to promote inhibitory interneuron function, including a subpopulation of cortistatin-expressing (Cort) neurons. This study identifies how TrkB depletion in Cort neurons impacts the Cort interneuron transcriptome as well as gene expression in the surrounding cellular milieu of mouse cortex. Our findings highlight TrkB-dependent molecular pathways in the maturation of inhibitory interneurons and further implicate BDNF signaling as critical for regulating excitatory/inhibitory balance. We identified BDNF regulation of a number of genes expressed in Cort neurons that are implicated in both autism and epilepsy, which is of note because these conditions are highly comorbid, and are hypothesized to share underlying molecular mechanisms.

## Introduction

Signaling of brain-derived neurotrophic factor (BDNF) via its transmembrane receptor tropomyosin receptor kinase B (TrkB) plays a significant role in the maturation and function of inhibitory neurons in the cortex and hippocampus (Yamada et al., 2002; Alcántara et al., 2006). Although inhibitory GABAergic interneurons represent only 10–15% of neurons in the rodent cortex (Meyer et al., 2011), they are highly heterogeneous, differing in morphology, firing patterns, response to neuromodulators, and molecular profiles (Tremblay et al., 2016). At least 26 different types of GABAergic interneurons have been identified in the hippocampus (Somogyi et al., 2004), and perhaps more in the cerebral cortex (Myers et al., 2007; Habib et al., 2017). Differences in firing properties, connectivity patterns, and molecular expression profiles are hypothesized to contribute to nonoverlapping functions of the respective classes.

BDNF–TrkB signaling plays an important role in the development of several classes of inhibitory interneurons. For example, BDNF regulates the differentiation and morphology of hippocampal interneurons (Marty et al., 1996), and BDNF deletion leads to reduction in several neuropeptide transcripts that define GABAergic populations, including somatostatin (SST), neuropeptide Y (NPY), substance P, and cortistatin (Cort) in the cortex (Glorioso et al., 2006; Martinowich et al., 2011). BDNF decreases the excitability of parvalbumin (Pvalb) interneurons in the dentate gyrus (Nieto-Gonzalez and Jensen, 2013), and accelerates their maturation in the visual cortex (Huang et al., 1999). While BDNF is expressed primarily in excitatory pyramidal neurons, but not in inhibitory interneurons, its receptor TrkB is widely expressed in both excitatory and inhibitory neurons (Cellerino et al., 1996; Gorba and Wahle, 1999; Swanwick et al., 2004). Levels of TrkB expression across different interneuron classes have not been explicitly quantified, but we previously reported that ∼50% of Cort-expressing interneurons express TrkB in the cortex (Hill et al., 2019).

Cortistatin is a secreted neuropeptide that is expressed in a distinct set of interneurons. This population partially overlaps with both Pvalb- and SST-expressing inhibitory interneurons, but its expression is seen prominently in the cerebral cortex and hippocampus (de Lecea et al., 1997). Cortistatin is similar in structure to SST and can bind all fived cloned somatostatin receptors (Veber et al., 1979; Csaba and Dournaud, 2001). However, Cort possesses some notably distinct functions, including its ability to induce slow-wave sleep activity (de Lecea et al., 1996) and regulated synaptic integration by augmenting the hyperpolarization-activated current *I*_H_ (Schweitzer et al., 2003). Cortistatin is expressed earlier than most inhibitory neuron markers in the brain, peaking at 2 weeks of age in rodents (de Lecea et al., 1997), which closely parallels the pattern of BDNF expression during neurodevelopment (Katoh-Semba et al., 1997). Reductions in BDNF signaling are associated with decreased expression of *Cort* transcripts (Martinowich et al., 2011; Guilloux et al., 2012), and conversely, the administration of cortistatin increases BDNF expression (Souza-Moreira et al., 2013). We previously demonstrated that TrkB expression in Cort interneurons is required to suppress cortical hyperexcitability. Specifically, mice in which TrkB is depleted in Cort interneurons develop spontaneous seizures and die ∼1 month after birth. Before developing seizures, these mice sleep for significantly less time and display hyperlocomotion (Hill et al., 2019). While this study established that TrkB signaling in Cort interneurons is critical to maintain appropriate levels of cortical excitability, the molecular mechanisms mediating Cort interneuron dysfunction downstream of TrkB signaling remain known.

To better understand the molecular mechanisms by which BDNF–TrkB signaling influences Cort interneuron development and function, we investigated the impact of TrkB deletion in these cells on the Cort interneuron transcriptome as well as gene expression in the surrounding cellular milieu. Translating ribosome affinity purification (TRAP) has been used to identify molecular profiles for many cell types in the mouse brain, including Cort interneurons (Doyle et al., 2008). Here, we used TRAP to assess how TrkB deletion impacts the molecular profile of these cells, and bulk RNA-sequencing (RNA-seq) to assess how this perturbation affects the surrounding milieu. Using this strategy, we identified several differentially regulated genes, including those encoding molecules important for calcium signaling as well as molecules that influence inhibitory/excitatory balance. Identification of the TrkB-dependent gene pathways that support Cort interneuron function contributes to our understanding of cortical hyperexcitability, which is important because changes in cortical excitability have been implicated in several brain disorders, including epilepsy and autism (Wang et al., 2013; van Diessen et al., 2015).

## Materials and Methods

### Animals

We selectively depleted TrkB in Cort-expressing cells by crossing mice in which Cre-recombinase is expressed under control of the endogenous *Cort* promoter (Cort^tm1(cre)Zjh^/J; referenced in text as Cort*^Cre^*; stock# 010910, The Jackson Laboratory; RRID:IMSR_JAX:010910; Taniguchi et al., 2011) to mice carrying a *loxP*-flanked TrkB allele (strain fB/fB, referenced in text as TrkB*^flox/flox^* (Grishanin et al., 2008; Baydyuk et al., 2011; Hill et al., 2019). Cort*^Cre^* mice were received from The Jackson Laboratory on a mixed C57BL/6J × 129S background. TrkB*^flox/flox^*mice were maintained on a C57BL/6J background. Cort*^Cre^*mice were backcrossed to a C57BL/6J background >12X, and TrkB*^flox/flox^*mice were backcrossed to a C57BL/6J background before initiating crosses.

For bulk homogenate RNA-seq experiments, the groups were postnatal day 21 (P21) Cort*^Cre^* or TrkB*^flox/flox^* (control group contained both genotypes) and Cort*^Cre^*;TrkB*^flox/flox^* (experimental group). As seizure onset begins at P21 (Hill et al., 2019), mice may have developed mild seizures by the time of brain extraction. In all RiboTag experiments, the RiboTag mouse (B6N.129-Rpl22^tm1.1Psam/J^; referenced in text as Rpl22^HA^; stock #011029, The Jackson Laboratory; RRID:IMSR_JAX:011029; Sanz et al., 2009) was used, which expresses a hemagglutinin (HA) tag on the ribosomal protein RPL22 (RPL22^HA^) under control of Cre-recombinase. For RiboTag experiments in Cort neurons, the groups were adult Cort*^Cre^*; Rpl22^HA^ Input versus Cort*^Cre^*; Rpl22^HA^ immunoprecipitation (IP). For RiboTag experiments in TrkB-deleted versus TrkB-intact Cort neurons, the groups were P21 Cort*^Cre^*; Rpl22^HA^ mice and Cort*^Cre^*;TrkB*^flox/flox^*;Rpl22^HA^ mice (experimental group).

All mice were housed in a temperature-controlled environment with a 12 h light/dark cycle and *ad libitum* access to standard laboratory chow and water. Mice were group housed based on genotype. All experimental animal procedures were approved by the SoBran Biosciences Institutional Animal Care and Use Committee. Male and female mice were included and analyzed for all experiments.

### RNA extraction and quantitative PCR

Mice were cervically dislocated, and cortices were flash frozen in isopentane. For bulk homogenate experiments in P21 control and Cort*^Cre^*;TrkB*^flox/flox^* mice, RNA was extracted using Life Technologies TRIzol (Thermo Fisher Scientific), purified using RNeasy minicolumns (Qiagen), and quantified using a Nanodrop spectrophotometer (Agilent Technologies). RNA concentrations were normalized and reversed transcribed using Life Technologies Superscript III (Thermo Fisher Scientific). Quantitative PCR (qPCR) was performed using a Realplex Thermocycler (Eppendorf) with Life Technologies GEMM mastermix (Thermo Fisher Scientific) and 40 ng of synthesized cDNA. Individual mRNA levels were normalized for each well to *Gapdh* mRNA levels. For validation of genes differentially expressed in control and experimental Ribotag samples, cDNA was synthesized using the Ovation RNA Amplification System V2 Kit (described below), and qPCR was performed as above. TaqMan probes were commercially available from Thermo Fisher Scientific (Gad1 Mm00725661_s1, Cort Mm00432631_m1, Gfap Mm01253033_m1, Wt1 Mm01337048_m1; Cxcr4 Mm01292123_m1; Calb1 Mm00486647_m1; Lgals1 Mm00839408_g1; Trpc6 Mm01176083_m1; Syt6 Mm04932997_m1; Gng4 Mm00772342_m1; Ttc9b Mm01176446_m1; S100a10 Mm00501458_g1; Nxph1 Mm01165166_m1; and Syt2 Mm00436864_m1; Gsn Mm00456679_m1) or as described in the study by Martinowich et al. (2011). Statistical analysis was conducted using GraphPad Prism (GraphPad Software). Comparisons between two groups were performed using unpaired Student’s *t* test. Data are presented as the mean ± SEM and statistical significance was set at *p < 0.05, **p < 0.01, ***p < 0.001, ****p < 0.0001.

### RNAscope single-molecule fluorescent *in situ* hybridization

Control and Cort*^Cre^*;TrkB*^flox/flox^* P21 mice were cervically dislocated and the brains were removed from the skull, flash frozen in isopentane, and stored at −80°C. Brain tissue was equilibrated to −20°C in a cryostat (Leica), and serial sections of cortex were collected at 16 μm. Sections were stored at −80°C until completion of the RNAScope assay. We performed single-molecule fluorescent *in situ* hybridization using the RNAscope Fluorescent Multiplex Kit version 2 [catalog #323100, Advanced Cell Diagnostics (ACD)] according to the study by Colliva et al. (2018). Briefly, tissue sections were fixed with a 10% neutral buffered formalin solution (catalog #HT501128, Sigma-Aldrich) for 20 min at room temperature and pretreated with protease for 20 min. Sections were incubated with commercially available *Wt1* (catalog #432711, ACD) and *Cre* (catalog #312281-C2, ACD) probes. Probes were fluorescently labeled with orange (excitation, 550 nm), green (excitation, 488 nm), or far red (excitation, 647) fluorophores using the Amp 4 Alt B-FL. Confocal images were acquired in z-series at 63× magnification using a Zeiss 700LSM confocal microscope. Images were blinded, and transcript colocalization was quantified using custom MATLAB functions. Briefly, cell nuclei were isolated from the DAPI channel using the cellsegm toolbox (Gaussian smoothening, adaptive thresholding, and splitting of oversized segmented nuclei; Hodneland et al., 2013). Once centers and boundaries of individual cells were isolated, an intensity threshold was set for transcript detection, and watershed segmentation was used to split detected pixel clusters in each channel into identified transcripts. Custom MATLAB functions were then used to determine the size of each detected transcript (regionprops3 function in Image Processing toolbox). Each transcript was then assigned to a nucleus based on its position in three dimensions. Transcripts with centers outside the boundaries of a nucleus were excluded from further analysis. A cell was considered to be positive for a gene if more than two transcripts were present.

#### Bulk cortex RNA-Seq

Cortices of control (*n* = 5) and Cort*^Cre^*;TrkB*^flox/flox^* (*n* = 5) mice were collected and flash frozen in isopentane. RNA was extracted from one hemisphere of each animal using Life Technologies TRIzol (Thermo Fisher Scientific), purified with RNeasy minicolumns (Qiagen), and quantified using Nanodrop. The Nextera XT DNA Library Preparation Kit was used to generate sequencing libraries according to manufacturer instructions. Samples were sequenced on the HiSeq2000 (Illumina).

### Ribotag and RNA-Seq of Cort interneurons

Cortices of Cort*^Cre^*; Rpl22^HA^ mice (*n* = 3) were collected and flash frozen in isopentane. For each sample (*n* = 3 Input, *n* = 3 IP), one hemisphere of the cortex from each animal was homogenized according to previously described protocols (Sanz et al., 2013). An aliquot of homogenate was flash frozen and reserved for “Input” samples. Ribosome-mRNA complexes (“IP” samples) were affinity purified using a mouse monoclonal HA antibody (MMS-101R, Covance; RRID:AB_2565334) and Pierce A/G magnetic beads (catalog #88803, Thermo Fisher Scientific). RNA from Input and IP samples was purified using RNeasy microcolumns (Qiagen) and quantified using the Invitrogen Ribogreen RNA Assay Kit (catalog #R11490, Thermo Fisher Scientific). Sequencing libraries were prepared using the SMARTer Stranded RNA-Seq Kit (Clontech) and sequenced on the HiSeq2000 (Illumina).

### Ribotag and RNA-Seq of Cort interneurons following disruption of BDNF–TrkB signaling

Cortices of control (*n* = 6) or Cort*^Cre^*;TrkB*^flox/flox^*;Rpl22^HA^ (*n* = 6) mice were collected and flash frozen in isopentane. One hemisphere of the cortex from each animal was homogenized according to previously described protocols (Sanz et al., 2013). Sixty-five microliters of total homogenate was flash frozen and reserved for Input samples. Ribosome-mRNA complexes (IP samples) were affinity purified using a mouse monoclonal HA antibody (MMS-101R, Covance) and PierceA/G magnetic beads (88803, Thermo Fisher Scientific). RNA from Input and IP samples was purified using RNeasy microcolumns (Qiagen) and quantified using the Invitrogen Ribogreen RNA Assay Kit (R11490 Thermo Fisher Scientific). The Ovation RNA Amplification System V2 Kit (7102, NuGEN) was used to amplify cDNA from 10 ng of RNA according to manufacturer instructions. cDNA was used for qPCR validation for *Cort* enrichment in IP versus Input samples. Sequencing libraries were generated with the Ovation SoLo RNA-seq System Mouse (0502–32, NuGEN) according to manufacturer instructions from 10 ng of RNA. Library concentration was quantified using the KAPA Library Quantification Kit (KR0405, KAPA Biosystems). Libraries were sequenced using the MiSeq Reagent Kit v3 (MS-102–3001, Illumina) and NuGEN Custom SoLo primer.

### RNA-Seq data processing and analyses

RNA-seq reads from all experiments were aligned and quantified using a common processing pipeline. Reads were aligned to the mm10 genome using the HISAT2 splice-aware aligner (Kim et al., 2015), and alignments overlapping genes were counted using featureCounts version 1.5.0-p3 (Liao et al., 2014) relative to Gencode version M11 (118,925 transcripts across 48,709 genes; March 2016). Differential expression analyses were performed on gene counts using the voom approach (Law et al., 2014) in the limma R/Bioconductor package (Ritchie et al., 2015) using weighted trimmed means normalization factors using the statistical models described below (Table 1). For each analysis, multiple testing correction was performed using the Benjamini–Hochberg approach to control for the false discovery rate (FDR; Kasen et al., 1990). Gene set enrichment analyses were performed on marginally significant genes using the subset of genes with known Entrez gene IDs against a background of all expressed genes using the clusterProfiler R Bioconductor package, which uses the hypergeometric test (Yu et al., 2012).

Cross-species enrichment analyses of the human SFARI (Banerjee-Basu and Packer, 2010) and Harmonizome (Rouillard et al., 2016) gene sets were performed with Fisher exact tests (which is identical to the above hypergeometric test on these 2 × 2 enrichment tables) on the subsets of homologous and expressed genes in each mouse dataset. For SFARI analyses, we considered the sets of (1) all genes in the mouse model database, (2) all genes in the human gene database (*N* = 1079 genes), (3) only genes that were syndromic or had gene scores of 1 or 2 (*N* = 235 genes, which correspond to high-confidence genes), and (4) only genes that had gene scores of 1 or 2, ignoring syndromic genes (*N* = 91 genes). All RNA-seq analysis code is available on GitHub: https://github.com/LieberInstitute/cst_trap_seq. Raw RNA-seq reads are available at BioProject Accession PRJNA602667.

#### Bulk cortex analysis for genotype effects

We used paired end read alignment and gene counting for these 10 samples (5 per genotype group). We analyzed 21,717 genes with reads per kilobase per million counted/assigned (RPKM normalizing to total number of gene counts, not mapped reads) >0.1. We performed differential expression analysis with limma voom using genotype as the main outcome of interest, further adjusting for the gene assignment rate (measured by featureCounts), the chrM mapping rate, and one surrogate variable.

#### Input versus IP analysis

We used paired end read alignment and gene counting for these six samples (three input and three IP). We analyzed 21,776 genes with RPKM > 0.1. We performed differential expression analysis with limma voom using fraction (IP vs Input) as the main outcome of interest, further adjusting for the gene assignment rate (measured by featureCounts) and also using the duplicateCorrelation function in limma to treat each mouse as a random intercept by using linear mixed-effects modeling.

#### IP analysis for genotype effects

We used single end read alignment and gene counting for these 12 samples (6 per genotype). We analyzed 21,187 genes with RPKM > 0.1. We performed differential expression analysis with limma voom using genotype as the main outcome of interest, further adjusting for the gene assignment rate (measured by featureCounts).

## Results

### Disruption of BDNF–TrkB Signaling in Cort Interneurons Alters Cortical Gene Expression

**Figure 1.**
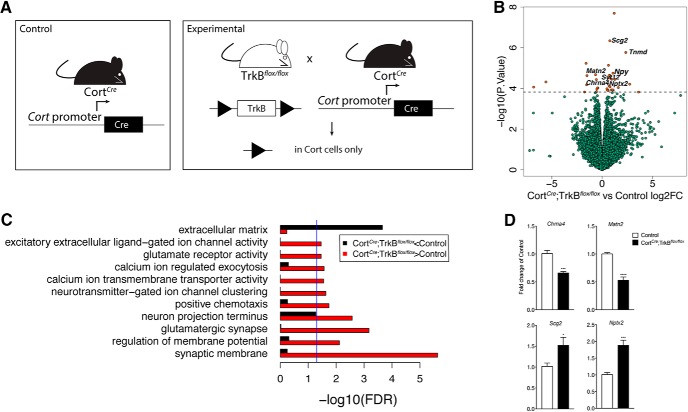
Loss of TrkB signaling in Cort interneurons causes gene expression changes in pathways regulating excitability. ***A***, Schematic of control and Cort*^Cre^*;TrkB*^flox/flox^* mice. ***B***, Volcano plot of bulk homogenate RNA-seq results with Cort*^Cre^*;TrkB*^flox/flox^* versus Control log_2_ fold change against −log_10_ *p* value. Orange dots represent genes that are significantly different in Cort*^Cre^*;TrkB*^flox/flox^* versus Control, including *Npy*, *Syt12*, *Nptx2*, and *Chrna4*. Green dots represent nonsignificant genes. See Extended Data Figure 1-1. ***C***, GO terms in the molecular function, biological processes, and cellular component categories for genes enriched and de-enriched in cortical tissue following ablation of TrkB in Cort neurons. See Extended Data Figure 1-2. ***D***, qPCR analysis validating genes found to be differentially expressed in bulk cortical homogenate RNA-seq of Cort*^Cre^*;TrkB*^flox/flox^* versus Control mice (*n* = 5 per genotype, Student’s unpaired *t* test; data are presented as the mean ± SEM: *p < 0.05 ***p < 0.001 ****p < 0.0001 vs control).

Mice with selective depletion of TrkB in Cort-expressing interneurons (Cort*^Cre^*;TrkB*^flox/flox^*; Fig. 1*A*) develop spontaneous seizures at approximately P21 (Hill et al., 2019). To better understand the molecular mechanisms downstream of TrkB signaling disruption in Cort interneurons that leads to hyperexcitability and disruption of excitatory/inhibitory balance, we performed bulk RNA-seq on the cortices of P21 Cort*^Cre^*;TrkB*^flox/flox^* (*n* = 5) and littermate controls (*n* = 5). Among the 21,717 expressed genes (at RPKM > 0.1), we identified 33 differentially expressed between Cort*^Cre^*;TrkB*^flox/flox^* and controls at FDR < 0.1 including 15 genes with absolute fold changes >2 (Fig. 1*B*, Extended data Fig. 1-1). Of particular interest, we observed increased expression of genes involved in cortical excitability such as tenomodulin (*Tnmd*, encoding an angiogenesis inhibitor implicated in Alzheimer’s; Tolppanen et al., 2011), *Npy* (encoding a neuropeptide synthesized by GABAergic interneurons; Karagiannis et al., 2009), and calsenilin (*Kcnip3*, encoding a calcium binding protein that influences cortical excitability; Pruunsild and Timmusk, 2005; 5.06-, 1.68-, and 1.52-fold changes, respectively; *p* = 1.7 × 10^−6^, 2.24 × 10^−5^, 1.26 × 10^−4^). We also observed decreased expression of ATPase plasma membrane Ca^2+^ transporting 4 (*Atp2b4)*, matrilin 2 (*Matn2*), and cholinergic receptor nicotinic alpha 4 subunit (*Chrna4*; 1.48-, 1.57-, and 1.49-fold changes, respectively; *p* = 1.46 × 10^−4^, 2.1 × 10^−5^, and 3.68 × 10^−5^, respectively). These genes encode proteins that are important for intracellular calcium homeostasis, formation of filamentous networks in the extracellular matrix, and acetylcholine signaling, respectively (Kuryatov et al., 1997; Mátés et al., 2002; Ho et al., 2015). To independently validate RNA-seq results, we confirmed differential expression of a subset of upregulated and downregulated genes using qPCR. We verified significant elevation of secretogranin II (*Scg2,*) and neuronal pentraxin II (*Nptx2*), additional genes of interest due to their roles in packaging neuropeptides into secretory vesicles and excitatory synapse formation, in Cort*^Cre^*;TrkB*^flox/flox^* mice compared with control mice (1.5- and 2-fold changes; *p* < 0.5 and 0.001, respectively; Fig. 1*D*; Ozawa and Takata, 1995; O’Brien et al., 1999). We also validated the reduction of *Chrna4* and *Matn2* transcripts (0.6- and 0.5-fold changes, *p* < 0.001 and 0.0001, respectively; Fig. 1*D*).

**Table 1:** Statistics

Figure	Data structure	Type of test	Type I error control	Notes
1*B*	Gene counts for differential expression analysis	Moderated *t* tests with linear regression (empirical Bayes)	FDR < 0.1	limma Bioconductor package: voom approach
1*C*	Gene set enrichment analysis	Hypergeometric test	FDR < 0.05	clusterProfiler Bioconductor package: compareClusters approach
1*D*	Normalized qPCR data	Student's *t* test	*p* < 0.05	
2*B*	Normalized qPCR data	Student's *t* test	*p* < 0.05	
2*C*	Gene counts for differential expression analysis	Moderated *t* tests with linear mixed effects modeling (empirical Bayes)	Bonferroni < 0.05	limma Bioconductor package: voom approach
2*D*	Gene set enrichment analysis	Hypergeometric test	FDR < 0.05	clusterProfiler Bioconductor package: compareClusters approach
2*E*	Normalized qPCR data	Student's *t* test	*p* < 0.05	
3*B*	Normalized qPCR data	Student's *t* test	*p* < 0.05	
3*C*	Differential expression analysis	Moderated *t* tests with linear regression (empirical Bayes)	FDR < 0.05	limma Bioconductor package: voom approach
3*D*	GO enrichment analysis	Hypergeometric test	FDR < 0.05	clusterProfiler Bioconductor package: compareClusters approach
4*A*	Normalized qPCR data	Student's *t* test	*p* < 0.05	
4*B*	Normally distributed	Student's *t* test	*p* < 0.05	

10.1523/ENEURO.0310-19.2019.f1-1 Figure 1-1 Differential gene expression analysis of Cort*^Cre^* vs Cort*^Cre^*;TrkB*^flox/flox^* bulk cortex for all expressed genes. Columns represent Symbol (mouse gene symbol), logFC (log2 fold change comparing experimental to control animals; positive values indicate higher expression in experimental animals), *t* [moderated *t* statistic (with empirical Bayes)], P.Value (corresponding *p* value from *t* statistic), adj.P.Val (Benjamini–Hochberg-adjusted *p* value to control the FDR), B (log odds of differential expression signal), gene_type (gencode class of gene), EntrezID (Entrez Gene ID), AveExpr [Average expression on the log2(counts per million + 0.5) scale], Length (coding gene length), and ensemblID (Ensembl gene ID). Download Figure 1-1, CSV file.

**Figure 2.**
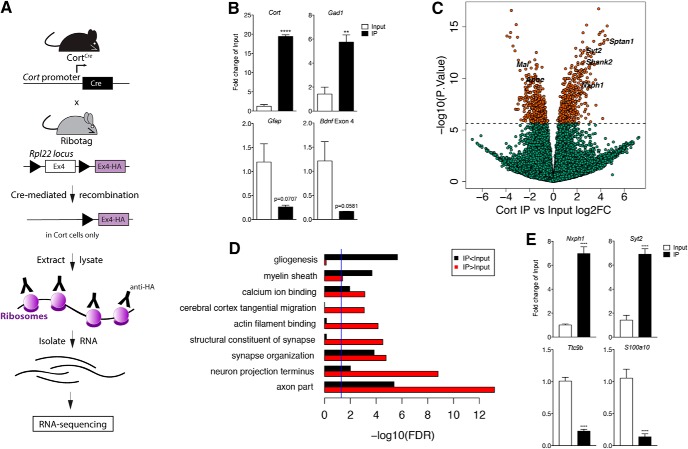
Translating ribosome affinity purification defines a unique molecular signature for cortistatin interneurons in the cortex. ***A***, Locus of the ribosomal protein Rpl22 in the RiboTag mouse and breeding strategy used to obtain Cort*^Cre^*;Rpl22*^HA^* mice. Schematic of RiboTag experimental workflow. ***B***, Validation of RiboTag allele expression in cortistatin neurons by qPCR for *Cort* as well as *Gad*, *Gfap*, and *Bdnf exon IV* (*n* = 3 per genotype, Student’s unpaired *t* test; data are presented as the mean ± SEM: **p < 0.01 ****p < 0.0001 vs control). ***C***, Volcano plot of RNA-seq results with Cort*^Cre^*;Rpl22*^HA^* Input versus Cort*^Cre^*;Rpl22*^HA^* IP log_2_ fold change against −log_10_ *p* value. Orange dots represent genes that are significantly different in Input versus IP fractions, including *Syt2*, *Nxph1*, *Mal*, and *Apoe*. Green dots represent nonsignificant genes. See Extended Data Figures 2-1 and 2-2. ***D***, GO terms in the molecular function, biological processes, and cellular component categories for genes enriched and de-enriched in Cort-expressing interneurons. See Extended Data Figure 2-3. ***E***, qPCR analysis validating genes found to be differentially expressed in Input versus IP RNA sequencing results (*n* = 3 per genotype, Student’s unpaired *t* test; data are presented as the mean ± SEM: ****p < 0.0001 vs control).

10.1523/ENEURO.0310-19.2019.f1-2 Figure 1-2 Gene ontology analysis of differentially expressed genes between Cort*^Cre^* and Cort*^Cre^*;TrkB*^flox/flox^* bulk cortex. Columns represent Cluster (label for set of differentially expressed genes), ONTOLOGY (gene ontology type: CC, cell compartment; BP, biological process; MF, molecular function), ID (gene ontology ID), Description (gene ontology set description), GeneRatio (fraction of differentially expressed genes were in the GO set), BgRatio (fraction of differentially expressed genes that were not in the GO set), Pvalue (*p* value resulting from hypergeometric test), p.adjust [Benjamini–Hochberg-adjusted *p* value (FDR)], qvalue (Storey-adjusted *p* value), geneID [gene symbols corresponding to the differentially expressed genes in the GO set (i.e., from GeneRatio above)], and Count [number of differentially expressed genes in the GO set (numerator of GeneRatio, to avoid forced Excel conversion to dates from some fraction)]. Download Figure 1-2, CSV file.

To identify signaling pathways impacted by differentially expressed genes in Cort*^Cre^*;TrkB*^flox/flox^* cortex compared with control, we performed gene ontology (GO) analysis on the subset of 269 marginally significant (at *p* < 0.005) genes with Entrez gene IDs, stratified by directionality (133 more highly expressed in control and 136 more highly expressed in Cort*^Cre^*;TrkB*^flox/flox^*; Fig. 1*C*, Extended Data Fig. 1-2). Consistent with the hyperexcitability phenotype in Cort*^Cre^*;TrkB*^flox/flox^* mice, analysis with the cellular component category showed terms such as glutamatergic synapse (*p* = 3.69 × 10^−5^), neuron projection terminus (*p* = 2.63 × 10^−4^), and collagen-containing extracellular matrix (*p* = 3.06 × 10^−8^). Analysis with the molecular function category showed terms such as calcium ion transmembrane transporter activity (*p* = 4.75 × 10^−4^), glutamate receptor activity (*p* = 1.35 × 10^−3^), and excitatory extracellular ligand-gated ion channel activity (*p* = 1.52 × 10^−3^). Analysis with the biological processes category showed terms such as positive chemotaxis (*p* = 3.07 × 10^−4^), calcium ion-regulated exocytosis (*p* = 6.67 × 10^−4^), and neurotransmitter-gated ion channel clustering (*p* = 4.55 × 10^−4^). Together, GO analysis of differentially expressed genes supports the hypothesis that the disruption of BDNF–TrkB signaling in Cort interneurons impacts signaling pathways that control excitatory/inhibitory balance and network excitability.

### Translatome profiling delineates a comprehensive molecular identity for Cort-expressing interneurons in the cortex

Given the critical role of Cort neurons in maintaining cortical excitatory/inhibitory balance, we sought to better understand the molecular profile of Cort neurons using TRAP followed by RNA-seq. We first crossed mice expressing Cre recombinase under control of the cortistatin promoter (Cort*^Cre^*) to mice expressing a Cre-dependent HA peptide tag on the RPL22 ribosomal subunit (Rpl22^HA^; Fig. 2*A*) to allow for HA tagging of ribosomes selectively in Cort neurons. Tagged ribosomes were immunoprecipitated (IP) from cortical homogenate tissue (Input) using an anti-HA antibody. Ribosome-associated RNA was isolated from IP samples and total RNA was isolated from Input samples.

10.1523/ENEURO.0310-19.2019.f2-1 Figure 2-1 Differential gene expression analysis of Cort*^Cre^* Input vs IP for all expressed genes. Columns represent Symbol (mouse gene symbol), logFC (log2 fold change comparing IP to Input samples; positive values indicate higher expression in IP samples), *t* [moderated *t* statistic (with empirical Bayes)], P.Value (corresponding *p* value from *t* statistic), adj.P.Val (Benjamini–Hochberg-adjusted *p* value to control the FDR), B (log odds of differential expression signal), gene_type (gencode class of gene), EntrezID (Entrez Gene ID), AveExpr [average expression on the log2(counts per million + 0.5) scale], Length (coding gene length), and ensemblID (Ensembl gene ID). Download Figure 2-1, CSV file.

10.1523/ENEURO.0310-19.2019.f2-2 Figure 2-2 CSEA of IP-enriched genes in Cort neurons. CSEA of IP-enriched genes identifies Cort interneurons. Bullseye plot of the output of CSEA reveals a substantial over-representation of Cort-positive neuron cell transcripts at multiple pSI levels among those transcripts (*n* = 100) found to be enriched in our IP samples from Cort neurons. Box highlights Cort-positive neurons. Download Figure 2-2, TIF file.

**Figure 3.**
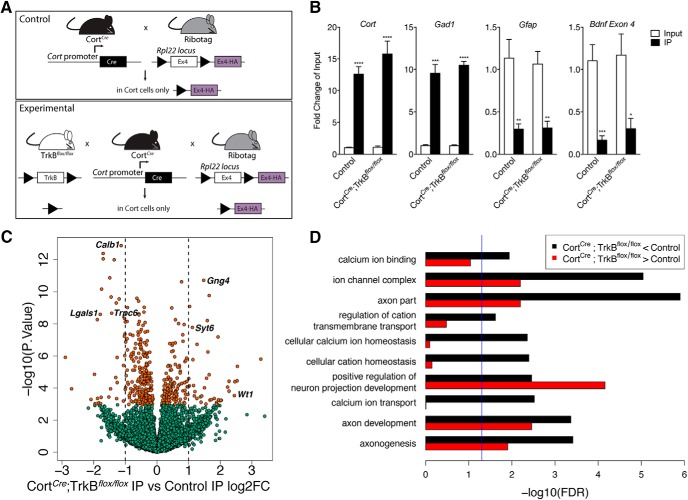
Loss of TrkB signaling in Cort neurons alters the expression of genes important for calcium homeostasis and axon development. ***A***, Breeding strategy used to obtain control Cort*^Cre^*; Rpl22^HA^ and experimental Cort*^Cre^*;TrkB*^flox/flox^*;Rpl22^HA^ mice. ***B***, Validation of Ribotag allele expression in cortistatin cells of control and Cort*^Cre^*;TrkB*^flox/flox^*;Rpl22^HA^ mice by qPCR of *Cort* as well as *Gad*, *Gfap*, and *Bdnf* exon IV (*n* = 6 per genotype, Student’s unpaired *t* test; data are presented as the mean ± SEM: *p < 0.05 **p < 0.01, ***p < 0.001, ****p < 0.0001 vs control). ***C***, Volcano plot of RNA-seq results with Cort*^Cre^*;TrkB*^flox/flox^*;Rpl22^HA^ IP versus Cort*^Cre^*;Rpl22^HA^ IP log_2_ fold change against −log_10_ *p* value. Orange dots represent genes that are significantly different in Cort*^Cre^*;TrkB*^flox/flox^*;Rpl22^HA^ IP versus Cort*^Cre^*; Rpl22^HA^ IP, including *Wt1*, *Calb1*, *Lgals1*, *Trpc6*, *Syt6*, and *Gng4*. Green dots represent nonsignificant genes. See Extended Data Figure 3-1. ***D***, GO terms in the molecular function, biological processes, and cellular component categories for genes enriched and de-enriched in Cort neurons following removal of TrkB and disruption of BDNF–TrkB signaling. See Extended Data Figures 3-2, 3-3, and 3-4.

10.1523/ENEURO.0310-19.2019.f2-3 Figure 2-3 Gene ontology analysis of differentially expressed genes between Cort*^Cre^* Input vs Cort*^Cre^* IP. Columns represent Cluster (label for set of differentially expressed genes), ONTOLOGY (gene ontology type: CC, cell compartment; BP, biological process; MF, molecular function), ID (gene ontology ID), Description (gene ontology set description), GeneRatio (fraction of differentially expressed genes were in the GO set), BgRatio (fraction of differentially expressed genes that were not in the GO set), Pvalue (*p* value resulting from hypergeometric test), p.adjust [Benjamini–Hochberg-adjusted *p* value (FDR)], qvalue (Storey-adjusted *p* value), geneID [gene symbols corresponding to the differentially expressed genes in the GO set [i.e., from GeneRatio above)], Count (number of differentially expressed genes in the GO set (numerator of GeneRatio, to avoid forced Excel conversion to dates from some fraction)]. Download Figure 2-3, CSV file.

Cell type-specific expression of the RiboTag allele in Cort neurons was confirmed by qPCR analysis showing significant enrichment of *Cort* transcripts in IP compared with the Input fraction (∼20-fold; *p* < 0.0001; Fig 2*B*). We also showed expected enrichment of glutamate decarboxylase 1 (*Gad1*) and depletion of glial fibrillary acidic protein (*Gfap)* and *Bdnf* exon IV-containing transcripts (6-, 0.25-, and 0.2-fold changes, *p* < 0.01, *p* = 0.0707, and *p* = 0.0581, respectively; Fig. 2*B*; Gorba and Wahle, 1999; Swanwick et al., 2004). Having confirmed successful IP from Cort-expressing interneurons, we generated stranded, ribosomal RNA depleted low-input libraries from Input and IP fractions and performed RNA-seq. Among the 21,776 expressed genes (at RPKM > 0.1), we identified 868 differentially expressed between Input and IP RNA fractions at Bonferroni-corrected *p* values <0.05 (and 5362 genes at FDR < 0.05), including 627 genes with absolute fold changes >2 (Fig. 2*C*, Extended Data Fig. 2-1). Reassuringly, differential expression analysis confirmed significant enrichment (IP/Input) of *Cort* transcripts (2.7-fold increase, *p* = 3.93 × 10^−3^) and significant depletion of transcripts for *Gfap, Mal* (T-cell differentiation protein), *Slc25a18* (solute Carrier Family 25 Member 18) and *Bdnf*, genes enriched in astrocytes, oligodendrocytes, and excitatory neurons, respectively (Schaeren-Wiemers et al., 1995b; Zhou and Danbolt, 2014; Hol and Pekny, 2015; Sasi et al., 2017). To independently validate our RNA-seq results, we confirmed differential expression of a subset of enriched and de-enriched genes, including neurexophilin 1 (*Nxph1*), synaptotagmin 2 (*Syt2*), tetratricopeptide repeat domain 9B (*Ttc9b*), and S100 calcium binding protein A10 (*S100a10*). These genes are of particular interest given their role in synapse function and calcium signaling (Pang et al., 2006; Svenningsson et al., 2013). Using qPCR, we verified significant enrichment of *Nxph1* and *Syt2* and de-enrichment of *Ttc9b* and *S100a10* in Cort IP compared with Input samples (7.0-, 7.0-, 0.2-, and 0.1-fold changes, respectively; *p* < 0.0001; Fig. 2*E*). To further confirm these results, we performed cell type-specific expression analysis (CSEA) on the top 100 differentially expressed genes based on fold change. This analysis confirmed significant over-representation of transcripts expressed in Cort neurons (Extended Data Fig. 2-2; Xu et al., 2014).

To discover the potential functional significance of the mRNAs enriched and depleted in Cort-expressing interneurons in the cortex, we performed GO analysis on the subset of 848 Bonferroni-significant genes differentially expressed in Cort IP compared with Input with Entrez gene IDs, stratified by directionality (440 more highly expressed in IP, 408 more highly expressed in Input; Fig. 2*D*, Extended Data Fig. 2-3). Genes enriched in IP fractions are involved in cellular component category terms such as axon part and neuron projection terminus and in biological process category terms such as synapse organization and cerebral cortex tangential migration. Genes enriched in Input fractions are involved in cellular component category terms such as myelin sheath and biological processes category terms such as gliogenesis. De-enrichment of myelin and gliogenesis pathways would be expected in the neuronal IP fractions, and hence further validate our approach.

### Loss of BDNF–TrkB signaling in Cort interneurons impacts genes critical for structural and functional plasticity

To better understand signaling pathways and cellular functions modulated by BDNF–TrkB signaling in Cort cells, we performed TRAP followed by RNA-seq in TrkB-depleted Cort interneurons. We intercrossed Cort*^Cre^*; Rpl22^HA^ mice to mice expressing a floxed TrkB allele (TrkB*^flox/flox^*) to allow for HA tagging of ribosomes in control Cort interneurons (Cort*^Cre^*; Rpl22^HA^) or TrkB-depleted Cort interneurons (Cort*^Cre^*;TrkB*^flox/flox^*;Rpl22^HA^; referred to hereafter as Cort^*Cre*^; TrkB^*flox/flox*^; Fig. 3*A*). For control and experimental animals (*n* = 6 each), tagged ribosomes were selectively immunoprecipitated (IP) from cortical homogenate tissue (Input) using an anti-HA antibody. Ribosome-associated RNA was isolated from IP samples and total RNA was isolated from Input samples.

10.1523/ENEURO.0310-19.2019.f3-1 Figure 3-1 Differential gene expression analysis of Cort*^Cre^* IP vs Cort*^Cre^*;TrkB*^flox/flox^* IP for all expressed genes. Columns represent Symbol (mouse gene symbol), logFC (log2 fold change comparing experimental to control animals; positive values indicate higher expression in experimental samples), *t* [moderated *t* statistic [with empirical Bayes)], P.Value (corresponding *p* value from *t* statistic), adj.P.Val (Benjamini–Hochberg-adjusted *p* value to control the FDR), B (log odds of differential expression signal), gene_type (gencode class of gene), EntrezID (Entrez Gene ID), AveExpr [average expression on the log2(counts per million + 0.5) scale], Length (coding gene length), and ensemblID (Ensembl gene ID). Download Figure 3-1, CSV file.

10.1523/ENEURO.0310-19.2019.f3-2 Figure 3-2 Gene ontology analysis of differentially expressed genes between Cort*^Cre^* IP vs Cort*^Cre^*;TrkB*^flox/flox^* IP. Columns represent Direction (+1 is upregulated in experimental compared with control, −1 is downregulated in experimental compared with control), Cluster (label for set of differentially expressed genes), ONTOLOGY (gene ontology type: CC, cell compartment; BP, biological process, MF, molecular function), ID (gene ontology ID), Description (gene ontology set description), GeneRatio (fraction of differentially expressed genes were in the GO set), BgRatio (fraction of differentially expressed genes that were not in the GO set), Pvalue (*p* value resulting from hypergeometric test), p.adjust [Benjamini–Hochberg-adjusted *p* value (FDR)], qvalue (Storey-adjusted *p* value), geneID (gene symbols corresponding to the differentially expressed genes in the GO set [i.e., from GeneRatio above]), and Count [number of differentially expressed genes in the GO set (numerator of GeneRatio, to avoid forced Excel conversion to dates from some fraction)]. Download Figure 3-2, CSV file.

10.1523/ENEURO.0310-19.2019.f3-3 Figure 3-3 Cort-enriched and TrkB-dependent genes in SFARI. Rows indicate SFARI genes (from either the human or mouse model databases, as described in the text) that were differentially expressed in at least one dataset (Cort*^Cre^* vs Cort*^Cre^*;TrkB*^flox/flox^* bulk cortex; Cort*^Cre^* Input vs IP; or Cort*^Cre^* IP vs Cort*^Cre^*;TrkB*^flox/flox^* IP). TRUE indicates that gene was significant in that particular χ^2^ enrichment test for that dataset and SFARI gene set. The first column indicates the Gencode ID. Download Figure 3-3, CSV file.

**Figure 4.**
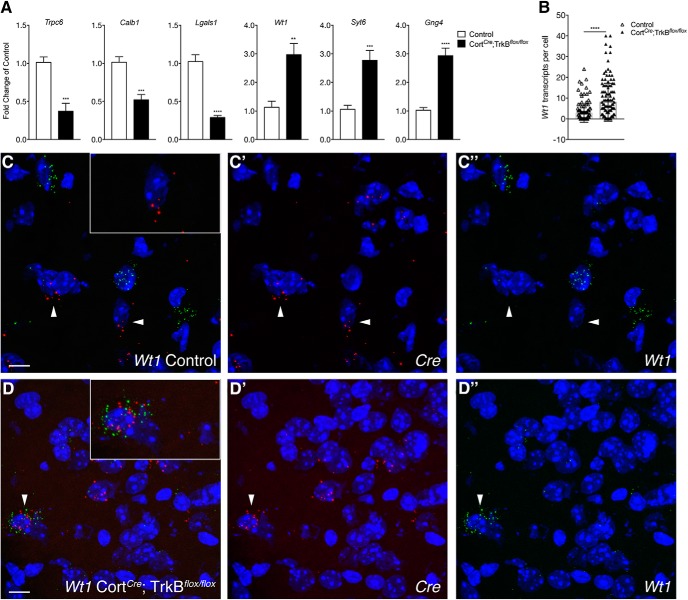
Validation of select targets from Control versus Cort*^Cre^*;TrkB*^flox/flox^* RNA-seq using qPCR and single-molecule fluorescence *in situ* hybridization. ***A***, qPCR analysis validating select genes (*Trpc6*, *Calb1*, *Lgals1*, *Wt1*, *Syt6*, *Gng4*) found to be differentially expressed in Cort*^Cre^*;TrkB*^flox/flox^* IP versus control IP RNA-seq data (*n* = 6 per genotype, Student’s unpaired *t* test; data are presented as the mean ± SEM: **p < 0.01, ***p < 0.001, ****p < 0.0001 vs control). ***B***, Quantification of *Wt1* transcripts in Cre positive cells of Cort*^Cre^*;TrkB*^flox/flox^* and control mice. ***C***, ***D***, Confocal *z*-projections of *Cre* and *Wt1* transcripts in the cortex from P21 Control (***C***) and Cort*^Cre^*;TrkB*^flox/flox^* (***D***) mice visualized with RNAscope *in situ* hybridization. *Wt1* transcripts (green) are more enriched in Cort neurons of Cort*^Cre^*;TrkB*^flox/flox^* than of Control mice. Inset depicts higher magnification of nuclei highlighted by arrows. Scale bars, ***C***, ***D***, 10 μm.

10.1523/ENEURO.0310-19.2019.f3-4 Figure 3-4 Cort-enriched and TrkB-dependent genes in Harmonizome database. Each Excel tab indicates the enrichment analyses from each disease gene set in the Harmonizome database. For “Bulk” and “IP genotype” tabs, columns represent Harmonizome disease set description, OR (odds ratio of being differentially expressed and in the disease set compared with being differentially expressed and not in the disease set), p.value (*p* value from χ^2^ test), adj.P.Val (Benjamini–Hochberg-adjusted *p* value), setSize (number of genes in the disease gene set), numSig (number of significantly differentially expressed genes in the gene set), ID (Harmonizome ID), and sigGenes [genes significantly differentially expressed and in the disease set (i.e. those genes driving the enrichment)]. For the “IP vs Input” tab, columns represent Harmonizome disease set description, Enrich_OR (odds ratios from genes significantly more highly expressed in Cort neurons than in Input), Enrich_Pval (corresponding *p* value from χ^2^ test), Deplete_OR (odds ratios from genes significantly more highly expressed in Input vs Cort neurons), Deplete_Pval (corresponding *p* value from χ^2^ test), and setSize (number of genes in the disease gene set). Download Figure 3-4, XLSX file.

Cell type-specific expression of the RiboTag allele in Cort interneurons was confirmed by qPCR analysis showing significant enrichment of *Cort* in IP versus Input fractions (12–15-fold; *p* < 0.0001; Fig. 3*B*). As expected, there was also significant enrichment of *Gad1* (8–12-fold; *p* < 0.001 for control, *p* < 0.0001 for Cort*^Cre^*;TrkB*^flox/flox^* mice; Fig. 3*B*) and significant depletion of *Gfap* (0.2–0.3-fold; *p* < 0.01 for both groups; Fig. 3*B*) and *Bdnf* exon IV-containing transcripts (0.1–0.3-fold; *p* < 0.001 for control, *p* < 0.05 for Cort*^Cre^*;TrkB*^flox/flox^* mice; Fig. 3*B*). We generated libraries from control and Cort*^Cre^*;TrkB*^flox/flox^* IP fractions and performed RNA-seq to generate a comprehensive molecular profile of genes enriched and depleted in TrkB-deficient Cort neurons. Among the 21,187 expressed genes (at RPKM > 0.1), we identified 444 differentially expressed between IP RNA fractions at FDR < 0.05, including 75 genes with fold changes >2 (Fig. 3*C*, Extended Data Fig. 3-1). Of particular interest, differential expression analysis confirmed significant enrichment (Cort*^Cre^*; TrkB*^flox/flox^* IP compared with control IP) of Wilms tumor 1 (*Wt1*), synaptotagmin 6 (*Syt6*), and G-protein subunit gamma 4 (*Gng4*) transcripts (5.45-, 2.17-, and 2.78-fold increase, *p* = 2.91 × 10^−4^, 1.68 × 10^−8^, 1.9 × 10^−11^, respectively). We observed significant depletion of transcripts for the transient receptor potential cation channel subfamily C member 6 (*Trpc6*), calbindin 1 (*Calb1*), and galectin 1 (*Lgals1*) transcripts (1.3-, 2.69-, 2.19-, and 3.46-fold decrease, *p* = 2.93 × 10^−8^, 2.13 × 10^−9^, 1.3 × 10^−13^, 2.55 × 10^−9^, respectively). Several of these genes are of interest due to their involvement in calcium signaling/homeostasis (Butz et al., 1999; Li et al., 2012; Schmidt, 2012) and axon development (Kobayakawa et al., 2015), pathways identified to be perturbed in bulk cortex following BDNF–TrkB disruption in Cort neurons (Fig. 1).

To explore the functional significance of the mRNAs enriched and depleted in Cort interneurons with disrupted BDNF–TrkB signaling, we performed GO analysis on the subset of 161 Entrez genes more highly expressed in TrkB-depleted Cort neurons and the 269 Entrez genes more highly expressed in control Cort neurons (Fig. 3*D*, Extended Data Fig. 3-2). Terms in both the molecular function and biological processes categories showed that cortistatin interneurons with disrupted BDNF–TrkB signaling were depleted for calcium ion binding, cellular calcium ion homeostasis, and calcium ion transport (FDR < 0.05). Ion channel complex and axon part, two cellular component category terms, were both depleted and enriched in Cort*^Cre^*;TrkB*^flox/flox^* interneurons, which indicates that disrupting BDNF–TrkB signaling modulates important cellular responses. For the biological process terms, positive regulation of neuron projection development and axon development were both enriched and depleted in those interneurons. Terms associated with both enrichment and depletion in Cort interneurons include different genes, which suggests that these cells may undergo gene-specific changes that support their ability to respond to different cellular signaling pathways. Together, these results support the hypothesis that BDNF–TrkB signaling regulates structural and functional plasticity in Cort interneurons to maintain excitatory/inhibitory balance.

To independently validate RNA-seq hits, we used qPCR to confirm significant enrichment and depletion of the above-mentioned transcripts in Cort*^Cre^*;TrkB*^flox/flox^* IP samples compared with control IP samples. We showed significant enrichment of *Wt1*, *Syt6*, and *Gng4* transcripts (3-, 2.8-, 2.9-fold changes, *p* < 0.01, 0.001, 0.0001, respectively). Furthermore, we showed significant depletion of *Trpc6*, *Calb1*, and *Lgals1* transcripts (0.3-, 0.5-, and 0.25-fold changes; *p* < 0.001, 0.001, 0.0001; Fig. 4*A*). We independently validated significant enrichment of *Wt1* using single-molecule fluorescence *in situ* hybridization in in TrkB-ablated Cort neurons (Cort*^Cre^*;TrkB^*flox/flox*^) compared with control Cort neurons (*p* < 0.0001; Fig. 4*B–D*).

### Genes important for Cort neuron identity and function overlap with those identified in autism spectrum disorder

Finally, we explored the potential clinical relevance of deficits in Cort neuron function using predefined genes sets from autism-sequencing studies [using Simons Foundation Autism Research Initiative (SFARI)] and disease ontologies (using Harmonizome). We found no significant enrichment in our bulk RNA-seq data with those identified in both autism spectrum disorder (ASD) and animal models relevant for ASD by the SFARI (Banerjee-Basu and Packer, 2010). However, we found enrichment for ASD genes among those genes highly expressed in Cort interneurons (Extended Data Fig. 3-3). For example, of the 239 genes in the “Mouse models” SFARI database expressed in our data, 27 (11.0%) were differentially expressed in Cort interneurons compared with total cortex, constituting a 6.3-fold enrichment (*p* = 9.25 × 10^−13^). Similarly, of the 937 genes in the “Human gene” SFARI database (which contains genes with rare variations associated with ASD from sequencing studies) with homologs expressed in our data, 93 were differentially expressed (9.9%, 4.2-fold enrichment; *p* = 9.03 × 10^−25^). These enrichments were preserved in the more stringent subset of ASD genes, either with [odds ratio (OR) = 5.71, *p* = 8.98 × 10^−14^] or without (OR = 6.55, *p* = 8.06 × 10^−8^) syndromic genes (see Materials and Methods). We further found significant enrichment for the overlap of genes differentially expressed in TrkB-depleted Cort cells compared with control Cort cells with those identified in both human and animal models of ASD as identified by SFARI (Extended Data Fig. 3-3). Here, of the 237 genes in the Mouse models SFARI database expressed in our data, 26 (11.0%) were differentially expressed in Cort*^Cre^*;TrkB*^flox/flox^* mice compared with control, constituting a sixfold enrichment (*p* = 6.85 × 10^−12^). Similarly, of the 917 genes in the Human gene SFARI database with homologs expressed in our data, 66 were differentially expressed (7.2%, 2.8-fold enrichment, *p* = 3.06 × 10^−11^), which were preserved in the smaller subset of more stringent ASD genes with (OR = 2.83, *p* = 0.002) or without (OR = 2.68, *p* = 0.02) syndromic genes (see Materials and Methods).

In addition to the overlap of ASD-relevant genes with those important for Cort identity and function, there was significant enrichment of many gene sets related to psychiatric disorders (at both the diseases and endophenotype levels) in the Harmonizome database (Rouillard et al., 2016) with those sets of genes preferentially expressed in Cort neurons and those dysregulated following TrkB depletion (Extended Data Fig. 3-4). In addition to enrichment for psychiatric disorders, we further found enrichment of epilepsy-related genes among TrkB-depleted and control Cort neurons (35 genes, *p* = 1.93 × 10^−12^; Extended Data Fig. 3-4). Together, these results further implicate Cort neurons in several debilitating human brain disorders (Xu et al., 2014).

## Discussion

### TrkB signaling in Cort interneurons regulates gene pathways that modulate cortical excitability

To better understand how disrupting BDNF–TrkB signaling in Cort cells impairs cortical function, we performed bulk RNA-seq on cortical tissue derived from Cort*^Cre^*;TrkB*^flox/flox^* and control mice and identified significant differential expression of genes important for excitatory neuron function. Pathway analysis of these differentially expressed genes revealed functions associated with glutamatergic synapses and synaptic membranes (Fig. 1*C*). For example, we observed altered expression of neuronal pentraxin II (*Nptx2*; log2FC = 1.26, *p* = 3.60 × 10^−5^), which encodes a synaptic protein implicated in excitatory synapse formation and neural plasticity (Gu et al., 2013) that is bidirectionally regulated by BDNF in hippocampal neurons both *in vitro* and *in vivo* (Mariga et al., 2015). Our dataset also shows increases in cAMP-responsive element Binding Protein 3 Like 1, which is necessary and sufficient to activate *Nptx2* transcription after BDNF treatment (Mariga et al., 2015). The protein encoded by *Nptx2* is also directly implicated in BDNF-mediated modulation of glutamatergic synapses, where it facilitates targeting and stabilization of AMPA receptors on excitatory synapses (Chang et al., 2010; Martin and Finsterwald, 2011; Pelkey et al., 2015). *Npy* is another differentially expressed gene (log2FC = 0.75, *p* = 2.24 × 10^−5^) that influences cortical excitability by reducing excitatory transmission onto neurons in the lateral habenula (Cheon et al., 2019) and inhibiting glutamatergic synaptic transmission in the hippocampus (Xapelli et al., 2008). NPY expression can slow the spread of seizures and has neuroprotective effects against excitotoxicity via increased BDNF signaling (Richichi et al., 2004; Xapelli et al., 2008).

*Bdnf* transcripts are paradoxically upregulated when comparing Cort*^Cre^*;TrkB*^flox/flox^* to controls in the bulk RNA-seq dataset (log2FC = 0.95, *p* = 3.55 × 10^−5^). Because TrkB receptors were selectively depleted from Cort interneurons, which do not synthesize BDNF (Gorba and Wahle, 1999; Swanwick et al., 2004), *Bdnf* increases likely result from upregulation in cortical excitatory neurons. *Bdnf* expression may be induced in excitatory neurons following the loss of TrkB in Cort interneurons for several reasons. First, in Cort*^Cre^*;TrkB*^flox/flox^* mice, impaired Cort interneuron function may facilitate disinhibition of excitatory neurons leading to increased cortical excitability and subsequent activity-induced *Bdnf* expression (Lu, 2003). Alternatively, increased *Bdnf* expression may be a compensatory mechanism attempting to counterbalance TrkB depletion in cortistatin cells. BDNF levels increase following seizures (Gall et al., 1991; Isackson et al., 1991; Mudò et al., 1996), and increases in BDNF can subsequently contribute to hyperexcitability and seizure propagation (Kokaia et al., 1995; Scharfman, 1997; Binder et al., 1999; Croll et al., 1999). Therefore, initiation and progressive worsening of seizures seen in Cort*^Cre^*;TrkB*^flox/flox^* mice could be exacerbated by increases in *Bdnf*. It should be noted that at the time of brain extraction (P21), mild seizures may have already have begun and could be influencing gene expression. In summary, depletion of TrkB receptors from Cort inhibitory interneurons may disrupt inhibitory signaling, leading to disinhibition of cortical excitatory neurons and disruption of network activity. This imbalance may push the cortex toward elevated excitation and increased expression of activity-regulated genes such as *Bdnf*, *Nptx2*, and *Npy*.

### Cortistatin neurons are enriched in genes relevant to ASD

Translatome profiling in Cort neurons showed enrichment of neuron-relevant genes such as *Syt2*, a synaptic vesicle membrane protein (Bornschein and Schmidt, 2018) and *Nxph1*, a protein important for dendrite–axon adhesion (Born et al., 2014). We also observed expected depletion of genes such as *Mal*, which is implicated in myelination (Schaeren-Wiemers et al., 1995a), and *Apoe*, which is synthesized in astrocytes (Holtzman et al., 2012). These data expand on a similar translatome profiling experiment previously performed by Doyle et al. (2008) using different mouse models and methodology. In that study, investigators used a mouse in which the EGFP-L10a ribosomal fusion protein is expressed under control of the *Cort* promoter in a bacterial artificial chromosome, and gene expression data were obtained using a microarray approach combined with TRAP. Here, we used a mouse that expresses Cre from the endogenous *Cort* promoter, and gene expression data were obtained using a Ribotag/RNA-seq approach. Reassuringly, there is significant overlap between the Doyle microarray dataset and our RNA-seq analysis (Extended Data Fig. 2-1).

Xu et al. (2014) showed candidate autism genes from human genetics studies are enriched in Cort cells, supporting the notion that cortical interneurons play a significant role in the etiology of ASD. Epilepsy, a common neurologic disorder characterized by recurrent seizures, is highly comorbid with ASD (Viscidi et al., 2013), and it has been proposed that these disorders may have overlapping genetic risk that points to shared underlying molecular and cellular mechanisms. Of note, interneuron dysfunction has been identified as a potential shared cellular mechanism in mouse models of both disorders (Jacob, 2016). Our results further demonstrate enrichment of genes associated with epilepsy and ASD in Cort neurons and highlight differential expression of several ASD and epilepsy genes in Cort neurons following the disruption of TrkB signaling. Our findings support the overlapping developmental origins of the two illnesses and highlight BDNF–TrkB signaling as potentially relevant to their etiology.

### Genes associated with calcium signaling and axonal development are disrupted following TrkB depletion in cortistatin interneurons

To identify putative molecular mechanisms that contribute to Cort interneuron dysfunction in Cort*^Cre^*;TrkB*^flox/flox^* mice, we compared the translatomes of intact Cort interneurons and Cort interneurons depleted of TrkB receptors. TrkB-depleted Cort neurons show dysregulation of genes associated with calcium ion homeostasis (*Calb1*, calcium binding protein; Schmidt, 2012) or calcium-dependent functions (*Syt6*, calcium dependent exocytosis; Fukuda et al., 2003), as well as genes associated with axon development (*Robo1*, axon guidance; Andrews et al., 2006) and cell–cell or cell–matrix interactions (*Lgals1*, plasma membrane adhesion molecule; Camby et al., 2006).

During development, cortical interneurons are generated in the ventral subcortical telencephalon and travel long distances to reach their final destination in cortical circuits, both tangentially from their birthplace in the ganglionic eminences and radially to their correct laminar position (Cooper, 2013). Chemokine signaling is important for the transition from tangential to radial migration, and the expression of chemokine receptors is directly affected by BDNF–TrkB signaling in the central nervous system, as well as in disease states such as cancer (Azoulay et al., 2018). We found that expression of *Cxcr4,* a chemokine receptor, is reduced in TrkB-depleted cortistatin interneurons by a factor of 4, which supports previous work showing modulation of CXCR4 expression and receptor internalization by BDNF–TrkB signaling (Ahmed et al., 2008). Degradation of this protein has been identified as a permissive signal for interneurons to leave tangential migratory streams (Sánchez-Alcañiz et al., 2011). Deletion of the gene leads to defects in cortical layer positioning (Li et al., 2008; Wang et al., 2011) and mutations result in premature accumulation of interneurons in the cortex. Although laminar distribution of Cort cells does not appear to be significantly altered by loss of TrkB (Hill et al., 2019), premature entry into the cortex may result in incorrect integration into the circuitry or improper axonal projections that cannot be inferred by laminar position. This explanation is further supported by altered expression of genes associated with axonogenesis, axon guidance, neuron projection terminus, and cell–matrix interactions (Fig. 3). The fact that CXCR4 is normally expressed in axons and functions to define their trajectory (Lieberam et al., 2005; Vilz et al., 2005; Miyasaka et al., 2007) provides additional strength to this hypothesis. In addition to *Cxcr4*, calcium signaling is important for stimulating (Behar et al., 1999) and halting (Bortone and Polleux, 2009) neuronal migration to the cortex, and Cort*^Cre^*;TrkB*^flox/flox^* mice show decreased expression of genes in calcium-related GO categories compared with control mice (Fig. 3), such as *Calb1*. Importantly, exogenous application of BDNF induces the elevation of intracellular calcium (Berninger et al., 1993; Marsh and Palfrey, 1996), and endogenous BDNF signaling elicits calcium responses at synapses (Lang et al., 2007). Additional work would be necessary to tease out the effects of interneuron migration, migratory stream maintenance, and correct development of projections during embryonic development in these mutant mice. An important future direction will be to evaluate the morphology of Cort neurons following the disruption of BDNF–TrkB signaling.

In summary, we provide evidence that the loss of BDNF–TrkB signaling in Cort interneurons leads to alterations in calcium signaling and axon development in these cells, which may contribute to altered excitatory/inhibitory balance in the cortex. Several of the genes enriched in Cort neurons and differentially expressed in TrkB-depleted neurons are implicated in both ASD and epilepsy. These data shed light on the role of BDNF–TrkB signaling in the function of Cort-expressing interneurons and provide the rationale for further functional studies of these interneurons.

## Synthesis

Reviewing Editor: Katalin Toth, Universite Laval Decisions are customarily a result of the Reviewing Editor and the peer reviewers coming together and discussing their recommendations until a consensus is reached. When revisions are invited, a fact-based synthesis statement explaining their decision and outlining what is needed to prepare a revision will be listed below. The following reviewer(s) agreed to reveal their identity: Joseph Dougherty.

Both reviewers agreed that the manuscript provides useful novel information and the study is carefully executed and the data analysis is appropriate. However, both reviewers identified several points where the manuscript could be improved. The authors should follow these constructive comments and revise the manuscript accordingly. These changes will not require new experiments.

Rev.#1.

This manuscript describes studies of gene expression in cortistatin (Cort)-expressing interneurons in the mouse cerebral cortex in the presence and absence of TrkB, the receptor for brain derived neurotrophic factor (BDNF). The authors use a transgenic mouse breeding strategy to selectively express the RiboTag/RPL22HA tool in Cort+ interneurons, which allows them to selectively map and measure ribosome-bound mRNAs in Cort+ cells. They then cross Cort-cre;RiboTag mice floxed TrkB mice, which allowed them to measure gene expression changes in Cort+ neurons in the presence and absence of TrkB. The authors confirm successful application of this strategy, and report several differentially expressed transcripts in Cort+ cells in the presence and absence of TrkB that indicate roles in excitatory/inhibitory balance in the neocortex.

This is a well-written manuscript that includes useful data to those studying cortical interneurons and/or gene expression in specific cell types. The methods and analyses are clear and rigorous. There are only a few minor concerns.

Minor points:

1) The title implies that the manuscript shows evidence of “modulation” of excitatory-inhibitory balance. Though the manuscript reports changes to synaptic- and excitability-related genes in Cort-cre x TrkB(flox/flox) mice, the evidence of modulation of excitatory-inhibitory balance appears only in previous studies (Hill et al, 2019), not here. It is recommended that “to modulate excitatory-inhibitory balance” should be removed from the title.

2) In the Introduction, cortistatin is described as being “restricted to the cerebral cortex and hippocampus” (p. 3, lines 2-3). Cort mRNA, however, appears to be found in many areas of the CNS, including olfactory bulbs (as can be seen in the Allen Institute ISH Atlas online). It is suggested that this be changed to “... its expression is seen prominently in the cortex and hippocampus”

3) The Methods section indicates that P21 mice were used. Given that the Cort-cre x TrkB(flox/flox) mice can experience seizures by this age (Hill et al, 2019), it should be noted, if possible, whether the mice included in these datasets had developed seizures by the time of brain extraction. If so, the potential impact of this variable on the differential gene expression analysis should be noted/discussed.

4) Throughout the methods section “cortex” is referred to (e.g., “cortices were flash frozen in isopentane”; p. 5, “RNA extraction and PCR”, sentence 1). Cortex is big and diverse, so It would be very useful for the authors to be more specific about the particular regions of cortex that were analyzed, imaged, etc.

5) Text annotations in differential gene expression figures (e.g. Figure 1b are too small to be read, even at considerable magnification on a computer screen (for this reviewer, 300% zoom was necessary for comfortable reading)

Rev.#2.

In this paper, the authors profile the molecular consequences of TrkB loss in Cort expressing cortical neurons using the TRAP methodology. The paper is a follow up to Hill 2019, where the authors report that loss to TrkB from Cort neurons leads to hyperexcitability, hyperlocomotion, lack of sleep, and eventual seizures and death. The paper makes several contributions: it identifies the gene expression changes in bulk RNAseq in cortices of mice with Cort specific TRAP be KO, it provides and in-depth analysis of Cort neuron gene expression in healthy mouse brain, and it characterizes the transcriptional/translational changes in those neurons following Cort KO. They find that there are many changes in these neurons (far more than can detected in bulk RNAseq) and that some of these overlap with known ASD genes. They also interpret these findings as being consistent with an altered Excitatory/Inhibitory imbalance

While there are some minor points that need clarification, generally the studies appear well executed in terms of appropriate statistical power, and the conclusions are kept largely within the scope of the data. I think it should be suitable for a short report at eNeuro with some revision.

Minor Concerns:

Authors described the depletion of Bdnf exon4 containing transcripts as expected when validating Cort TRAP samples. Is there a reference to go along with this assertion?

The authors did not confirm loss of TrkB in Cort neurons here. However, examination of a prior study (Hill 2019) show that it has been confirmed with this same mouse cross. That should be referenced here.

It is hard for this reviewer to know how to critically evaluate the statement in the discussion attributed to “Anonymous 2019” that the layer positions of the Cort neurons is not different with TrkB mutation. Is this a paper that we have been blinded to for double-blind peer review? Or a personal communication from an anonymous source? It is odd and I don't know what to do with it. This data appears to be in the Hill paper that they cite in the introduction. Perhaps they should just cite that here too and avoid the mystery?

Page 9. “Supporting the notion that loss of TrkB expression in these cells causes a disruption in excitatory/inhibitory balance,..” I didn't follow why these particular genes were related to e/I imbalance? Authors should better support this claim using the literature, or just remove the statement.

Page 14 - what are the GO terms for the downregulated transcripts? Does the decrease in axonal gene expression indicate that there are less elaborate axons or other changes in morphology? The authors discuss the possibility that the neurons mis-migrate without Trkb signaling. Is it worth also discussing whether the Cort neurons might have a stunted development in terms of altered morphology? Is this something they can evaluate in fluorescently labeled Cort neurons with a sholl or similar analysis?

In addition, it may be worth giving more background on what is known about this class of neurons in the introduction.

Page 15 - Overlap with SFARI db: The authors should repeat this analysis also stratifying for SFARI gene score. In the current iteration of the SFARI db, there are now rankings to define the confidence with which these genes are genetically associated with autism. Some of the lower ranking genes have very little evidence they are associated with ASD. Also, please describe which stat is being used to assign a p-value to fold enrichment here or in methods.

Please clarify whether the qPCR replications are done on independent samples and that N given corresponds to independent biological replicates (i.e. # of mice). Please confirm that the data meet assumptions for a T-test. If not, either conducting the stats on CT values, or using a rank test might be more appropriate and sensitive.

Typos:

In methods, why do the authors sometimes use C57BL/6, C57BL/6J and sometimes C57B16/J in the writing?

The phrase: “P21 Cort Cre HA; Rpl22 mice (control group contained both genotypes)” is a bit confusing. It is clear why the authors need to parentheses in the para above, but not the second time.
