# Supplementary figures and images for: TrkB Signaling Influences Gene Expression in Cortistatin-Expressing Interneurons
Source: eNeuro. 2020 Feb 6;7(1):ENEURO.0310-19.2019. doi: 10.1523/ENEURO.0310-19.2019 (PMC7031852; doi:10.1523/ENEURO.0310-19.2019)

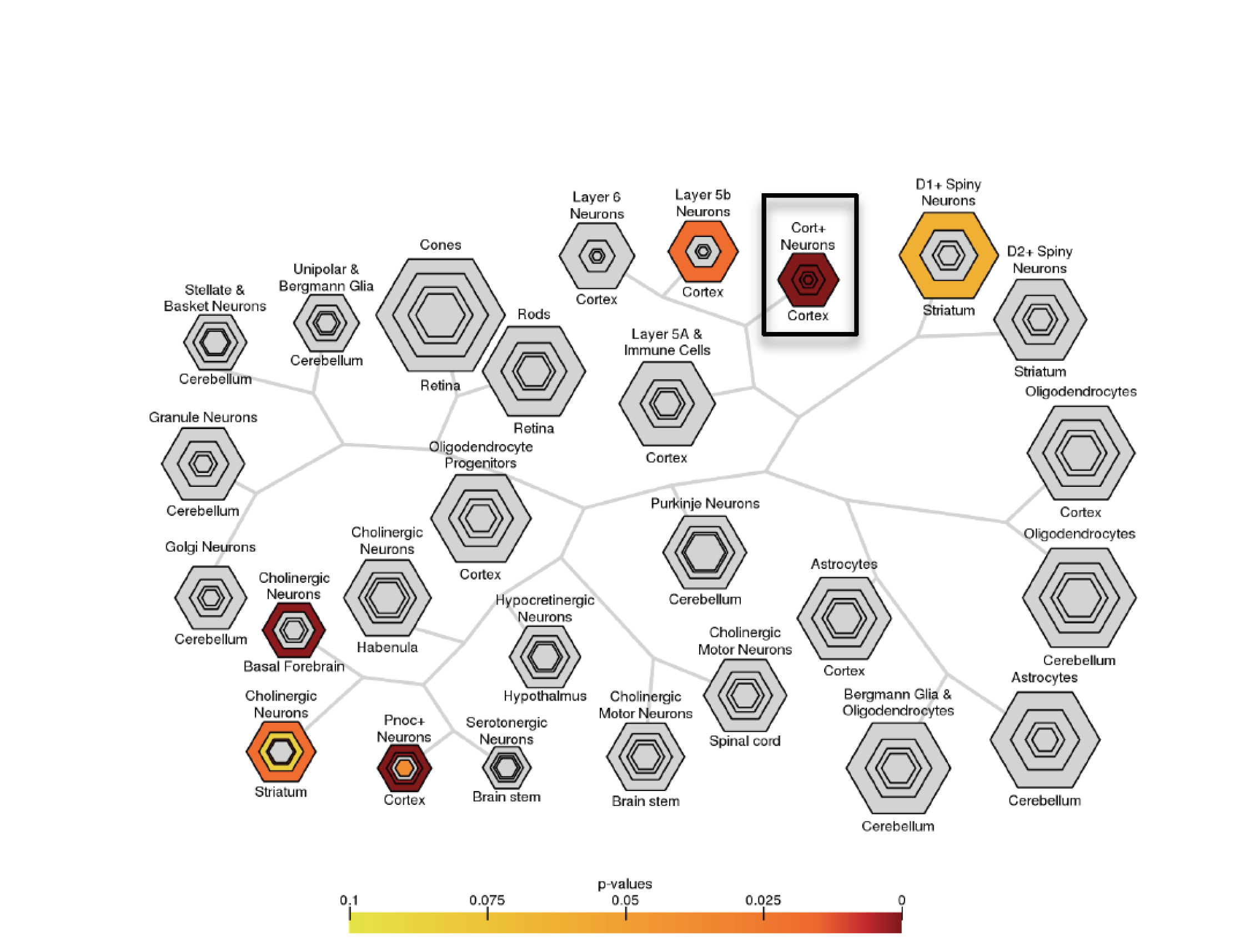

Supplement: Figure 2-2 — CSEA of IP-enriched genes in Cort neurons. CSEA of IP-enriched genes identifies Cort interneurons. Bullseye plot of the output of CSEA reveals a substantial over-representation of Cort-positive neuron cell transcripts at multiple pSI levels among those transcripts (n = 100) found to be enriched in our IP samples from Cort neurons. Box highlights Cort-positive neurons. Download Figure 2-2, TIF file. [file sup_enu-eN-NWR-0310-19-s04.tif]
